# Clays enhanced with niobium: potential in wastewater treatment and reuse as pigment with antibacterial activity

**DOI:** 10.3762/bjnano.16.13

**Published:** 2025-02-10

**Authors:** Silvia Jaerger, Patricia Appelt, Mario Antônio Alves da Cunha, Fabián Ccahuana Ayma, Ricardo Schneider, Carla Bittencourt, Fauze Jacó Anaissi

**Affiliations:** 1 Federal University of Technology - Paraná - UTFPR, Campus Toledo, Rua Cristo Rei, 19. 85902-490, Toledo, Brazil; 2 Chemistry Department, Universidade Estadual do Centro-Oeste, Guarapuava 85040-167, PR, Brazil; 3 Department of Chemistry, Universidade Tecnológica Federal do Paraná, Pato Branco 85503-390, Brazil; 4 Chimie des Interactions PlaBEa-Surface (ChIPS), Research Institute for Materials Science and Engineering, University of Mons, 7000 Mons, Belgium

**Keywords:** adsorption, bentonite, hybrid pigment, niobium, photocatalysis, water remediation

## Abstract

Bentonite clay sourced from the Guarapuava region, Brazil, was modified with niobium oxide (BEOx) and niobium phosphate (BEPh) to act as an adsorbent and photocatalyst in the remediation of wastewater containing methylene blue (MB) dye. Additionally, colored materials were evaluated for their potential as antibacterial hybrid pigments. The bentonite clay modified with niobium was prepared by a solution containing swelling clay mixed with niobium oxide (NbOx) and niobium phosphate (NbPh) in a water solution; after that, the suspension was calcinated. X-ray diffractometry, X-ray photoelectron spectroscopy, and laser-induced breakdown spectroscopy assessed the modifications induced by the incorporation of niobium compounds into the clay, confirming the presence of niobium in the bentonite clay. Following characterization, the BEOx and BEPh samples were used as adsorbents or photocatalysts for treating solutions containing the MB dye (400 mg·L^−1^) at 25 °C. The results showed adsorption and photocatalysis efficiency above 94% for both samples. The blue-colored BEOx and BEPh samples were then applied as a hybrid pigment. The power pigment and its dispersion in colorless paint were evaluated by the CIEL*a*b* color space, and the Δ*E* parameters show values above 12, indicating a very strong color parameter difference. Subsequently, the efficacy of BEOx and BEPh as a hybrid pigment was assessed using the minimum inhibitory concentration (MIC) assay against two bacteria strains: *Bacillus cereus* (ATCC 10876) and *Proteus mirabilis* (ATCC 35649). The analysis revealed remarkable antibacterial activity against *Proteus mirabilis*, suggesting a preferential selectivity for Gram-negative bacteria.

## Introduction

The most found dye pollutants in wastewater on a global scale originate from textile, plastic, paper, food, cosmetics, mineral, and pharmaceutical industries, among others, resulting in significant environmental impacts [1]. Dyes, as chemical compounds that impart color to different materials, play a crucial role in industries requiring coloring, such as textile, food, cosmetics, rubber, printing, paper, and plastic. Globally, an estimated 7 × 10^5^ tons of dyes are produced, with 10–15% typically disposed of as wastewater pollutants [2]. Among the most used dyes, methylene blue (MB) is an intense blue cationic dye important in medical sciences, chemistry, and biology, as well as widely used in the textile industry [2]. Prolonged exposure to MB can result in adverse health effects, including abdominal disorders, respiratory distress, skin sensitization, and blindness [3]. The dark blue color of MB in wastewater reduces light penetration into aquatic organisms, disturbing the balance of the ecosystem and harming various forms of life [3]. Effluents and water bodies containing MB require prioritized treatment due to its adverse effect on water quality. Therefore, it is crucial to explore remedial strategies for MB, especially considering the water scarcity challenges that many countries face [3].

To satisfy environmental regulations, a range of wastewater treatment technologies with inherent advantages and limitations are available, encompassing processes such as advanced oxidation, extraction, and biodegradation [4]. Unfortunately, these methods exhibit inefficiencies due to the generation of secondary pollution and high operational costs. Biological and anaerobic degradation of dyes may yield carcinogenic by-products [4–5], highlighting the significant challenge in purifying water contaminated with dyes necessitating the development of cost-effective technologies for their removal from industrial effluents.

Adsorption emerges as a widely used method for pollutant removal from wastewater due to its design simplicity, operational ease, and relatively straightforward regeneration of the adsorbent. Various adsorbents including chitosan, cellulose, organophilic clays, kaolinite and montmorillonite clays, and activated carbon have been used for removing toxic compounds from polluted water [6]. Among these adsorbents, bentonite and smectite clays exhibit advantageous properties as an adsorbent, characterized by their low cost, abundant availability, nontoxic nature, and large surface area [2,7]. Additionally, its negatively charged surface renders it favorable for the adsorption of cations [7]. Bentonite clay is abundantly found in Guarapuava, Paraná, Brazil. This natural clay has predominantly the bentonite phase (at least 50%), known as montmorillonite. Isomorphic substitution of cations between the interlayer space of montmorillonites by exchanging Na^+^, Ca^2+^, Mg^2+^, and Cu^2+^ cations add other functionalities to the resulting material [7].

Heterogeneous photocatalysis is a cost-effective alternative to biological treatment methods for purifying polluted water [8]. Using semiconductors as heterogeneous catalysts proves to be more efficient than traditional methods, as the photocatalytic process gradually decomposes contaminating molecules without generating residues from the original organic matter, thus avoiding the disposal of sludge [8]. This approach allows the removal of various organic pollutants, including textile dyes, using solid semiconductors (e.g., NbOPO_4_ and Nb_2_O_5_) and photons (with energy greater than the bandgap energy of the semiconductor) to generate OH^•^ radicals (strong oxidants), leading to the mineralization of organic pollutants, including textile dyes [8].

In this study, the strategy and objective are to modify bentonite clay with niobium phosphate and niobium oxide by the calcinated method, and then use it as an adsorbent or photocatalyst to treat MB dye solutions and reuse this material as a hybrid pigment. Considering the semiconductor properties of niobium and the high capacity of the clay to remove pollutants from wastewater, we proposed in this research to use the niobium-modified clay as an adsorbent and photocatalyst to treat MB dye solutions. To reuse this colored material, recovered from the adsorption and photocatalysis tests, we propose using these as-prepared, blue-colored samples as a hybrid pigment. Subsequently, the as-prepared, blue-colored samples were evaluated for their color properties by CIEL*a*b* color space as a pigment in the powder form and dispersed in colorless paint. The novel-developed pigments, specifically the smectite clay modified with niobium-containing adsorbed dye, were investigated as antibacterial pigments.

## Experimental

### Materials

The bentonite clay from the Guarapuava region in the Parana State, Brazil, was purchased from a local supplier. Niobium phosphate (NbOPO_4_) and niobium pentoxide (Nb_2_O_5_) were provided as donations by Companhia Brasileira de Metalurgia e Mineração (CBMM). Methylene blue, with molecular mass of 319.8513 g·mol^−1^, was obtained from Nuclear (Brazil).

### Clay modified with niobium

**Figure 1:**
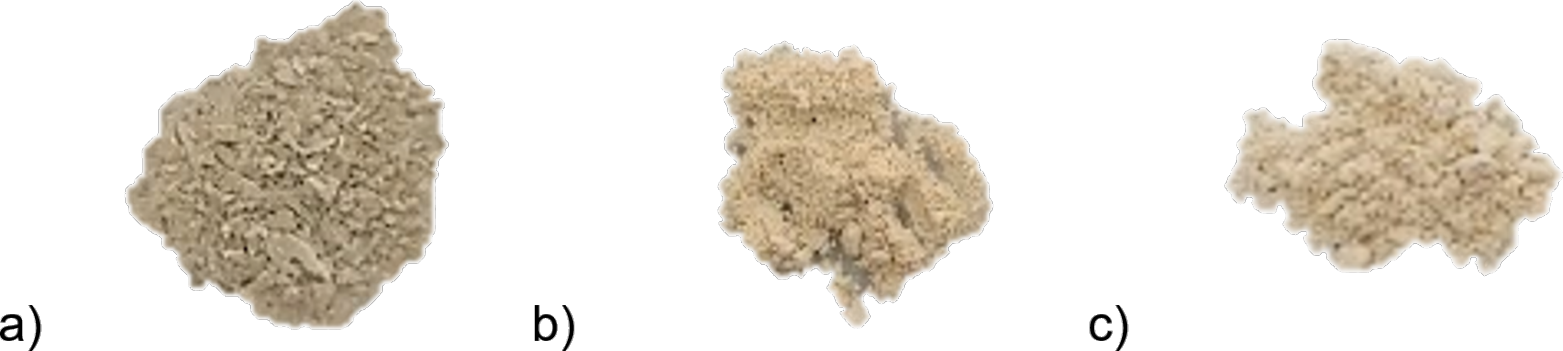
The appearance of the sample of bentonite: raw bentonite (a); bentonite modified with NbOPO_4_ (b), and bentonite modified with Nb_2_O_5_ (c).

First, the clays were swollen; for this purpose, 2 g of bentonite clay was dispersed in 100 mL of water, and the resulting suspension was kept under stirring for 24 h. In this experiment step, the color of raw clay was brown (Figure 1a). Then, 3.14 g of NbOPO_4_ and Nb_2_O_5_ was added. The clay/Nb suspension was continuously stirred for 72 h at 65 °C. The color of the bentonite modified with niobium changes to light yellow (Figure 1b,c). Finally, after being cooled to room temperature, the suspensions were subjected to thermal treatment at 500 °C, with a heating rate of 5 °C/min. These samples were named BEPh and BEOx for modification with NbOPO_4_ and Nb_2_O_5_, respectively.

### Adsorption and photocatalysis tests

Before the adsorption and photocatalysis assessments, a stock solution of the MB dye was prepared at a concentration of 1 g·L^−1^. The calibration analytical curve was established using a UV–vis spectrophotometer at a wavelength of 664 nm.

Adsorption experiments were conducted in batches containing 250 mg of the BEPh and BEOx samples under agitation at 25 °C. A volume of 100 mL of MB solutions at a concentration of 400 mg·L^−1^ were used for 3 h. The adsorption experiment was carried out considering ambient light conditions in the laboratory. Following the adsorption process, the clay/Nb samples were centrifuged at 3500 rpm for 10 min, and the final concentration of the solutions was determined using a UV–vis spectrophotometer. These samples were named A-BEPh and A-BEOx to designate the modification with NbOPO_4_ and Nb_2_O_5_, respectively.

Photocatalytic tests were performed using 100 mL of an MB solution at a concentration of 400 mg·L^−1^. This experiment used 250 mg of the BEPh and BEOx samples as catalysts. The experimental setup system included a thermostatic Pyrex glass reactor at 25 °C (open), a magnetic stirrer, and a UV lamp (253.7 nm, 15 W, 220 V) within a dark chamber. After 3 h of exposure, the solutions were centrifuged at 3500 rpm for 10 min, and their final concentrations were determined using a UV–vis spectrophotometer. These samples were labeled as A-BEPhP and A-BEOxP to designate the modifications with NbOPO_4_ and Nb_2_O_5_, respectively.

The adsorption efficiency of MB by the clays was calculated using Equation 1:


[1] $$\%\text{Remotion}=100\cdot \frac{\left(C_{0}-C_{\text{f}}\right)}{C_{0}},$$

where *C*_0_ (mg·L^−1^) is the initial concentration of the solution, and *C*_f_ (mg·L^−1^) is the concentration of the solution after the adsorption experiment.

The efficiency of MB photodegradation (*X*%) was determined by Equation 2:


[2] $$X\left(\%\right)=\frac{\left(M_{0}-M_{\text{f}}\right)}{M_{0}}\cdot 100,$$

where *M*_0_ and *M*_f_ are the concentrations of MB at the beginning and at the end of the photocatalytic test, respectively.

### Dispersion of the pigments clay/Nb and clay/Nb/MB in colorless commercial paint

The samples A-BEPh, A-BEOx, A-BEPhP, and A-BEOxP were separated through centrifugation and dried in an oven at 70 °C. The clay powders, clay/Nb, and clay/Nb/MB powders were tested as pigments in colorless commercial paint. For this purpose, a 10% (w/w) ratio of the pigments in colorless commercial paint (a transparent paint) was used. A sodium hydroxide solution (NaOH, 1 mol·L^−1^) was dripped onto the clay powders until reaching a pH between 8 and 10. Subsequently, this suspension was blended with the transparent paint. Plaster molds were painted with both colorless and pigmented paint. After the paint dried, the color was characterized through colorimetric analysis (CIEL*a*b*) and UV–vis spectroscopy.

### Antimicrobial activity test

The antimicrobial properties of the BEPh, BEOx, A-BEPh, A-BEOx, A-BEPhP, and A-BEOxP samples were investigated against the bacteria *Bacillus cereus* (ATCC 10876) (Gram-positive) and *Proteus mirabilis* (ATCC 35649) (Gram-negative). The samples of bentonite clay modified with niobium were dispersed in water. The assay followed the protocols described by the Clinical and Laboratory Standards Institute (CLSI). The samples were evaluated using the minimum inhibitory concentration (MIC) method [9–10] at concentrations ranging from 1.25 to 0.09 mg/mL.

The bacterial stock cultures were activated by culturing in brain heart infusion (BHI) broth at 37 °C for 24 h. Then, the cellular concentrations were standardized according to the McFarland 0.5 scale (≈1.5 × 10^8^ CFU/mL) using a spectrophotometer at a wavelength of 625 nm in saline water tubes. Subsequently, 100 µL of Mueller–Hinton broth was added to all wells in 96-well plates, followed by duplicate addition of the samples using serial microdilution, and finally, 10 µL of the inoculum. The plates were then incubated for 24 h at 37 °C. After incubation, 20 µL of the TTC dye (0.125% w/v - 2,3,5-triphenyltetrazolium chloride 0.125%) (NEON®) was added to all wells, and the plate was kept in an oven for an additional two hours. The antibacterial activity was determined by MIC, observing the presence/absence of viable bacteria due to the reaction of the TTC dye with the enzyme succinate dehydrogenase (present in the mitochondria), leading to the formation of a salt called Formazan with a pink-reddish color.

### Characterization

X-ray diffraction (XRD) measurements of the powder were conducted using a Rigaku BEartLab SE 3 kW diffractometer equipped with Cu Kα radiation (λ = 1.5410 Å) operating at 40 kV and 30 mA. Data were collected in scanning mode in steps between 4° and 75° (2θ) with a step size of 0.05°/s. The basal distance was obtained using Bragg's Law.

Fourier-transform infrared spectroscopy (FTIR) spectra were collected on a Perkin Elmer Frontier FTIR spectrometer using KBr pellets containing 1% by weight of the samples. Analyses of all samples were performed in the range of 4000 to 400 cm^−1^ with a resolution of 4 cm^−1^, accumulating 10 scans.

Absorbance measurements of the supernatant solutions were analyzed using a UV–vis spectrophotometer (UV-1800 SHIMADZU) with a 1 cm path length glass cuvette at λ_max_ (maximum absorbance) in nanometers.

Samples collected after the adsorption process had the electronic spectra analyzed using an Ocean Optics USB-2000 instrument for solid samples with a tungsten lamp in the range of 200–800 nm in diffuse reflectance mode.

Powder and paint-applied samples were analyzed by colorimetry, based on the CIEL*a*b* system, using a portable colorimeter (NR60CP – 3NH).

The oxidation state and elemental composition of the samples were evaluated using X-ray photoelectron spectroscopy (XPS) with a PHI Genesis instrument from Physical Electronics (Chanhassen, MN, USA), equipped with a monochromatic Al Kα X-ray source. The binding energy was calibrated based on the C 1s peak at 284.6 eV.

Laser-induced breakdown spectroscopy (LIBS) analyses were carried out by using the Applied Spectra J200 equipment. The clay samples were pelletized in circular discs of 1.2 cm and approximately 0.2–0.3 cm of height. The discs were prepared using 5 mg of each sample, and then they was pressed at 10 tons. The spectra were collected under air atmosphere between 186 and 1050 nm using a laser line at 50% as the source performing 10 shots per spot with 50 μm of diameter and gate delay of 0*.*5 μs.

A static laser scattering (SLS) Horiba LA-960 equipment assessed the powder particle size distribution measurements using a 15 mL cuvette accessory and water as the dispersion medium. The refractive index was set to 1.640 for red and blue lines.

## Results and Discussion

**Figure 2:**
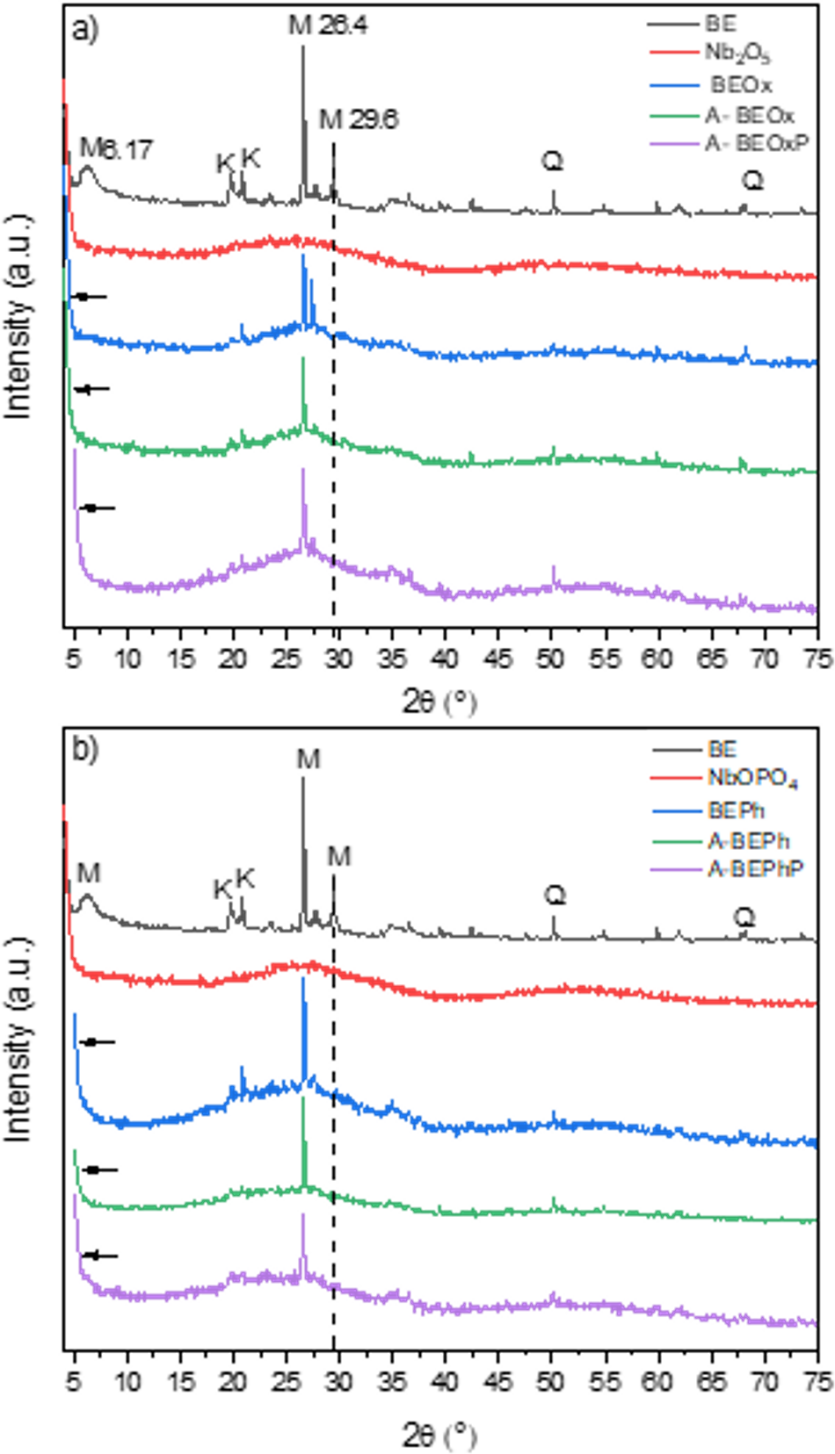
X-ray diffraction patterns of the bentonite samples and those modified with niobium oxide (a) and niobium phosphate (b).

The XRD profile for bentonite clay, niobium phosphate, niobium oxide and its modifications with niobium phosphate and niobium oxide (BEPh and BEOx, respectively), as well as the samples obtained after adsorption/photocatalysis of MB (A-BEPh, A-BEOx, A-BEPhP, A-BEOxP), are shown in Figure 2. The XRD analysis for bentonite before modification with niobium indicates dioctahedral montmorillonite (M-COD 9002779 M*_x_*(Al_4−_*_x_*Mg*_x_*)Si_8_O_20_(OH)_4_) with an amount of kaolinite (K-COD 1011045 Al_2_H_4_O_9_Si_2_) and quartz (Q-COD 9012600 SiO_2_) at 13.8%, 41.6%, and 44.6%, respectively [8].

The characteristic reflections of montmorillonite (M) for the basal spacing correspond to approximately 15 Å (d001 = 14.88 Å), related to the interlayer distance of 2:1 clays, resulting in a spacing between 14.0–15.0 Å corresponding to the peak at 6.17 2θ (^o^) [11]. The quartz phase (Q) was identified by the presence of reflections at 2θ = 50.02° and 68.32°. The characteristic peaks that allowed the identification of the kaolinite phase (K) were at 2θ = 12.48°and 20.14° [7].

Figure 2a,b presents diffraction patterns with amorphous characteristics of the niobium compounds NbOPO_4_ and Nb_2_O_5_. Two broad peaks were identified, one at approximately 25.54° (2θ) and the other at 49.73° (2θ) [12–13]. The amorphous pattern characteristic of niobium compounds was maintained upon modification of the bentonite clay with these compounds.

**Figure 3:**
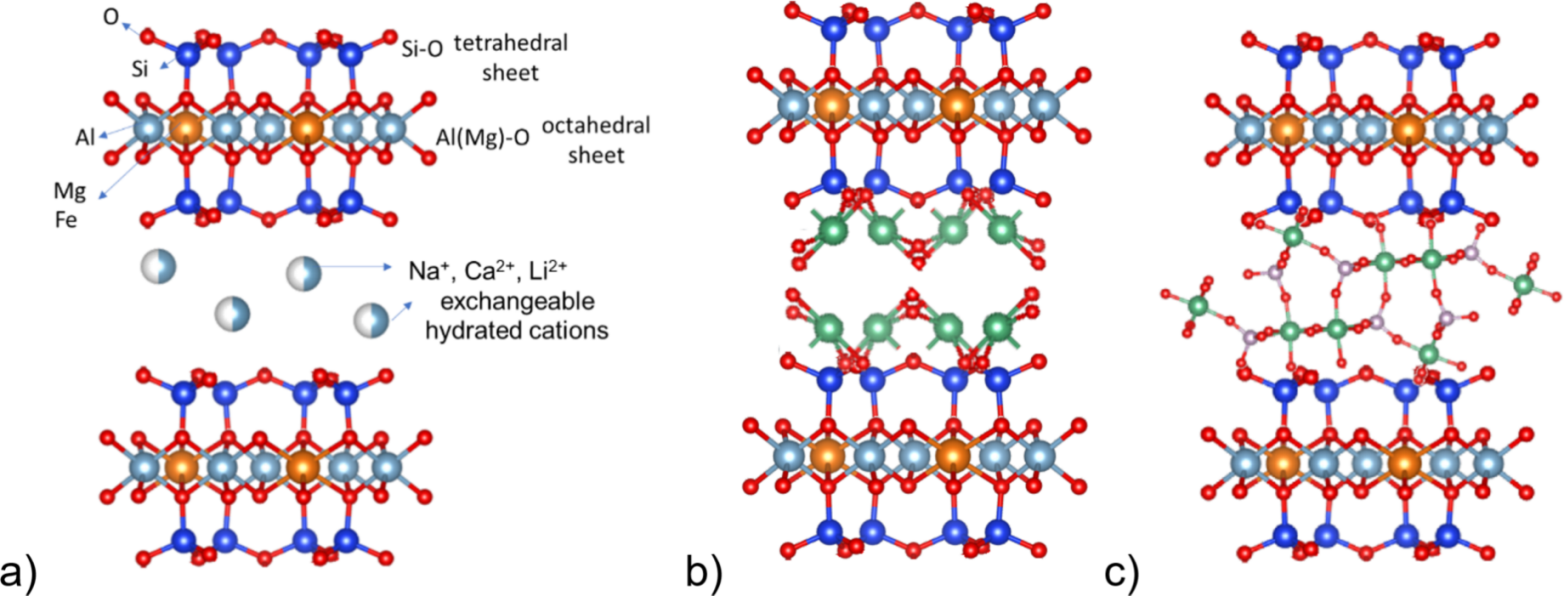
Schematic representation of bentonite clay (a), bentonite modified with NbOX (BeOx) (b), and bentonite modified with NbPh (BePh) (c). The structures were obtained with the Vesta program using the COD 1100106, COD 1534619, and COD 1517684 for montmorillonite, niobium oxide, and niobium phosphate, respectively.

The X-ray pattern of the samples of bentonite clay modified with the niobium compounds (BeOx and BEPh) and the same obtained after adsorption/photocatalysis of MB (A-BEPh, A-BEOx, A-BEPhP, and A-BEOxP) show the characteristic diffraction peak at approximately 26.4° (2θ) indicating that the niobium modification did not destroy the structure of the montmorillonite layer [14]. However, the relative intensity of the diffraction peak at 29.6° (2θ) decreased with the addition of the niobium compounds. This decrease can be attributed to the intercalation of the niobium in the interlayer of the clay, which increases the layer distance and the ion coordination of the interlayer to produce the polyhydroxyniobium. Another fact that can explain this result is the slight shifts of the montmorillonite characteristic diffraction peak (6.17° 2θ) to lower 2θ angles as observed in Figure 2a,b, suggesting increased interlayer space of the clay. The studies of Qiu et al. (2019) [14] and Gallo et al. (2006) [15] observed the same results, and indicated that the bentonite clay modified with niobium initiated the inorganic pillarization process from the polyhydroxyniobium process [14–15]. The supposed structure of bentonite clay modified with niobium phosphate and niobium oxide proposed in this study can be observed in Figure 3.

**Figure 4:**
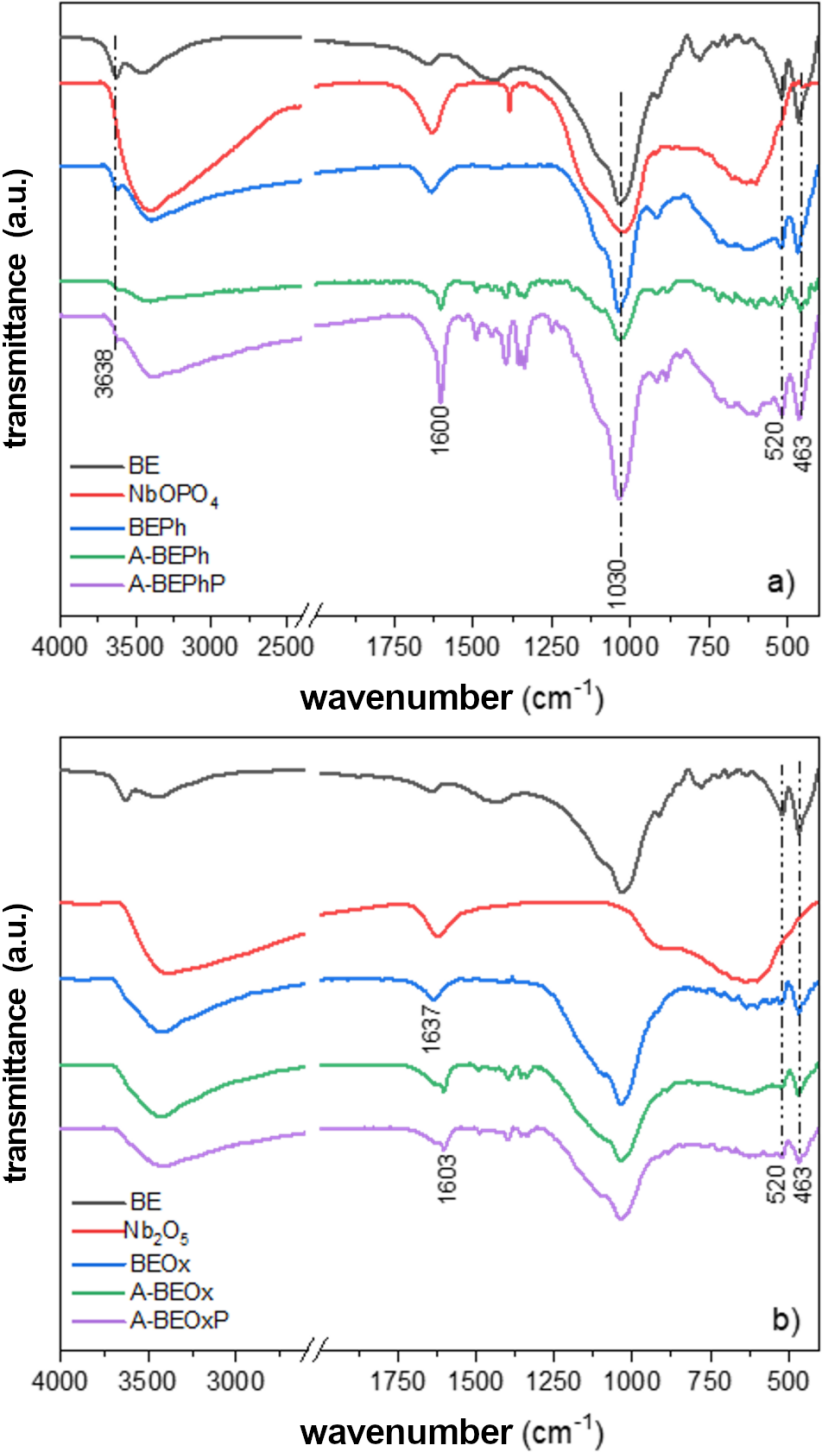
FTIR spectra for the bentonite samples modified with niobium phosphate (a) and niobium oxide (b).

The FTIR spectra of the bentonite before and after modification with niobium are shown in Figure 4. It can be observed that for the BE samples and all those modified with NbOPO_4_ (Figure 4a), the spectra show a narrow band in the region of 3638 cm^−1^, associated with the (Al–OH–Al) Al_2_OH vibrational stretching, indicative of bentonite with a high aluminum content in octahedra [7,16]. The spectra for the NbOPO_4_ samples and for the bentonite modified with niobium phosphate (BEPh) before and after adsorption/photocatalysis (Figure 4a) show a band in the region of 1043 cm^−1^, due to the vibrational mode (ν) of asymmetric stretching of the phosphate ion.

The broad band in the region of 3385 cm^−1^ and the narrow band in the region of 1600 or 1637 cm^−1^ present in the spectrum for BE and the clay samples modified with NbOPO_4_ and Nb_2_O_5_ are attributed to the stretching of hydroxyl and angular deformation of the water molecule, respectively [7,17]. The band in the region of approximately 1115 cm^−1^ is related to the stretching vibrations of Si–O. Bands in the region of 520 and 463 cm^−1^, which appear in all spectra of bentonite and bentonite modified with niobium, are attributed to the stretching and bending of Si–O present in the clay layers [7].

The spectra for Nb_2_O_5_ and NbOPO_4_ and the bentonite modified with niobium before and after adsorption/photocatalysis present an intense band in the region of 630 cm^−1^ related to the stretching of the Nb–O bond. The A-BEPh, A-BEPhP, A-BEOx, and A-BEOxP samples exhibit a set of bands in the region from 1477 to 1277 cm^−1^ typical for the identification of the MB dye, indicating the presence of the dye in the clay structure [18].

**Figure 5:**
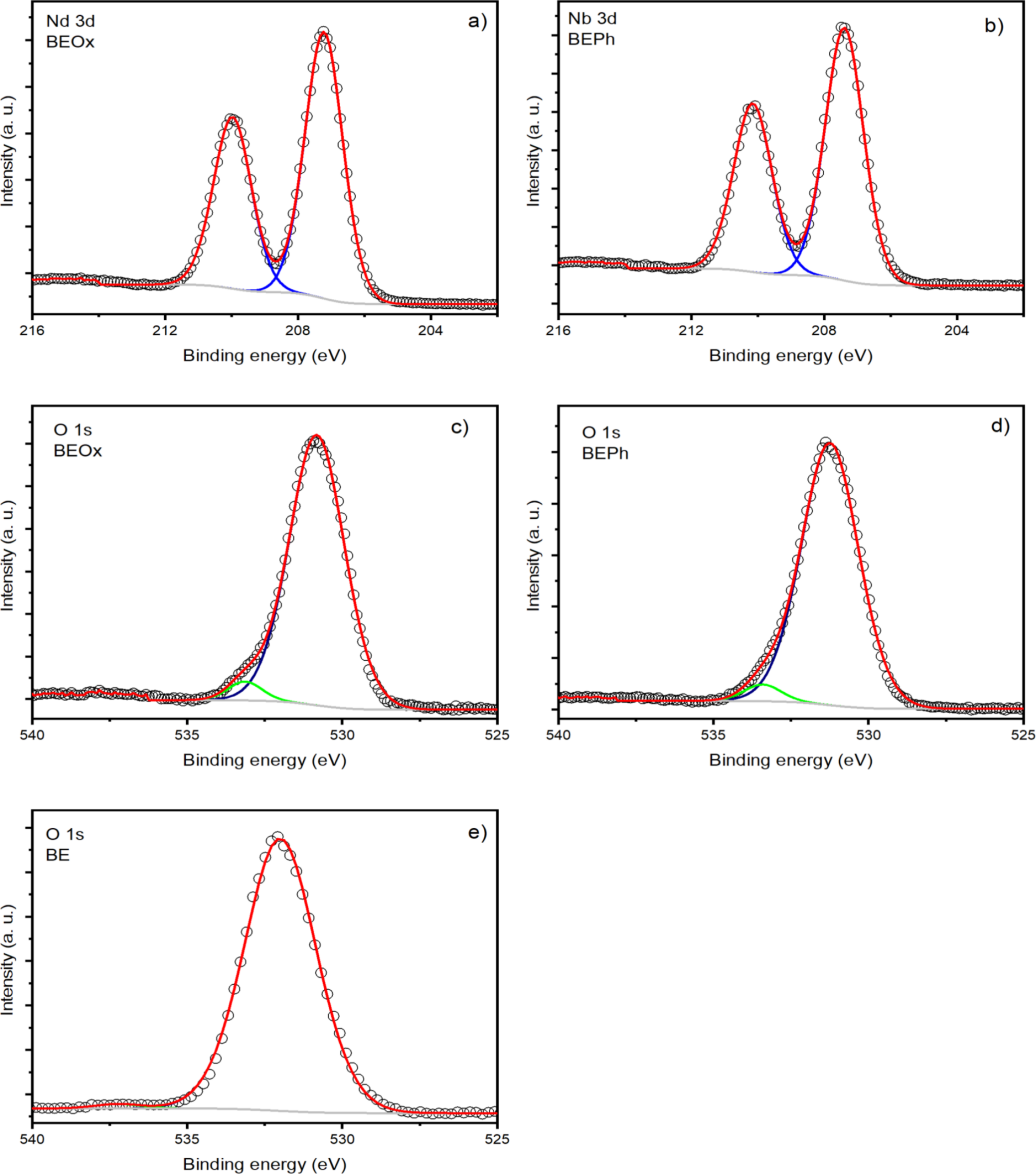
Photoelectron spectroscopy results for Nb3d (a and b for the samples BEOx and BEPh, respectively), O 1s (c, d, and e for the samples BEOx, BEPh, and BE, respectively).

Figure 5 shows the XPS analysis of niobium in BEOx and BEPh samples (Figure 4a and Figure 4b, respectively). The Nb 3d spectra exhibit two distinct peaks centered at 207.5 and 210.2 eV, corresponding to Nb 3d_5/2_ and Nb 3d_3/2_, respectively, indicative of niobium +5. The O 1s XPS spectra are shown in Figure 5c for BEOx and Figure 5d for BEPh. The spectra of samples BEOx and BEPh are reproduced with two components centered at 530.9 and 533.5 eV. The component centered at 531.0 eV can be attributed to photoelectrons emitted from oxygen atoms in Si–O, Al–O, or Nb–O bonds, whereas the low-intensity component at a higher binding energy can be associated with the hydroxyl OH^−^ group of Nb–OH located in the interlayer region of the clay. These results confirm the increased interlayer space of the clay observed in DRX results (Figure 2). Figure 5e shows the O 1s spectrum recorded on sample BE. The high-intensity component centered at 532.0 eV is associated with oxygen bonds in Si–O–Si bonds [12].

Table 1 presents the chemical compositional analysis of the samples BE, BEOx, and BEPh determined by XPS. The results indicate that the bentonite clay modified with niobium phosphate (BEPh) exhibits a phosphorous content of 2.0% (wt %), thereby confirming the successful modification of the clay with this niobium compound. Furthermore, the samples BEOx and BEPh displayed niobium relative concentrations of 6.4 wt % and 4.0 wt %, respectively. These results suggest the incorporation of niobium into the clay matrix. In Table 1, considering the montmorillonite phase with the empirical formula M*_x_*(Al_4−_*_x_*Mg*_x_*)Si_8_O_20_(OH)_4_, when it was modified with the niobium compounds, the element sodium had its percentage decreased compared with that in the bentonite sample. However, the little change in the percentage of aluminum and silica, corresponding to the octahedral and tetrahedral layer, respectively, confirms that the niobium compounds are exchanged with sodium counter ions.

**Table 1:** Composition of the samples BE, BEOx, and BEPh determined by XPS.

Samples	wt %											
	C	N	O	F	Na	Mg	Al	Si	P	Ca	Nb	Fe

BEOx	6.4	–	62.5	1.1	2.6	2.1	4.8	13.1	–	1.0	6.4	–
BEPh	7.9	1.5	62.7	1.0	1.7	1.3	4.4	12.5	2.0	1.0	4.0	–
BE	9.3	–	55.0	–	6.0	3.8	6.5	17.5	–	1.4	–	0.5

In recent years, laser-induced breakdown spectroscopy, an optical emission spectroscopic technique, has emerged as a rapid qualitative and quantitative analysis [19]. This spectroscopic technique can be explained by the short-duration, high-intensity pulsed laser being focused on a material, producing a plasma called laser-induced plasma (LIP). Qualitative and quantitative information about a sample is obtained by measuring the spectral delivery of the laser-induced plasma [19].

**Figure 6:**
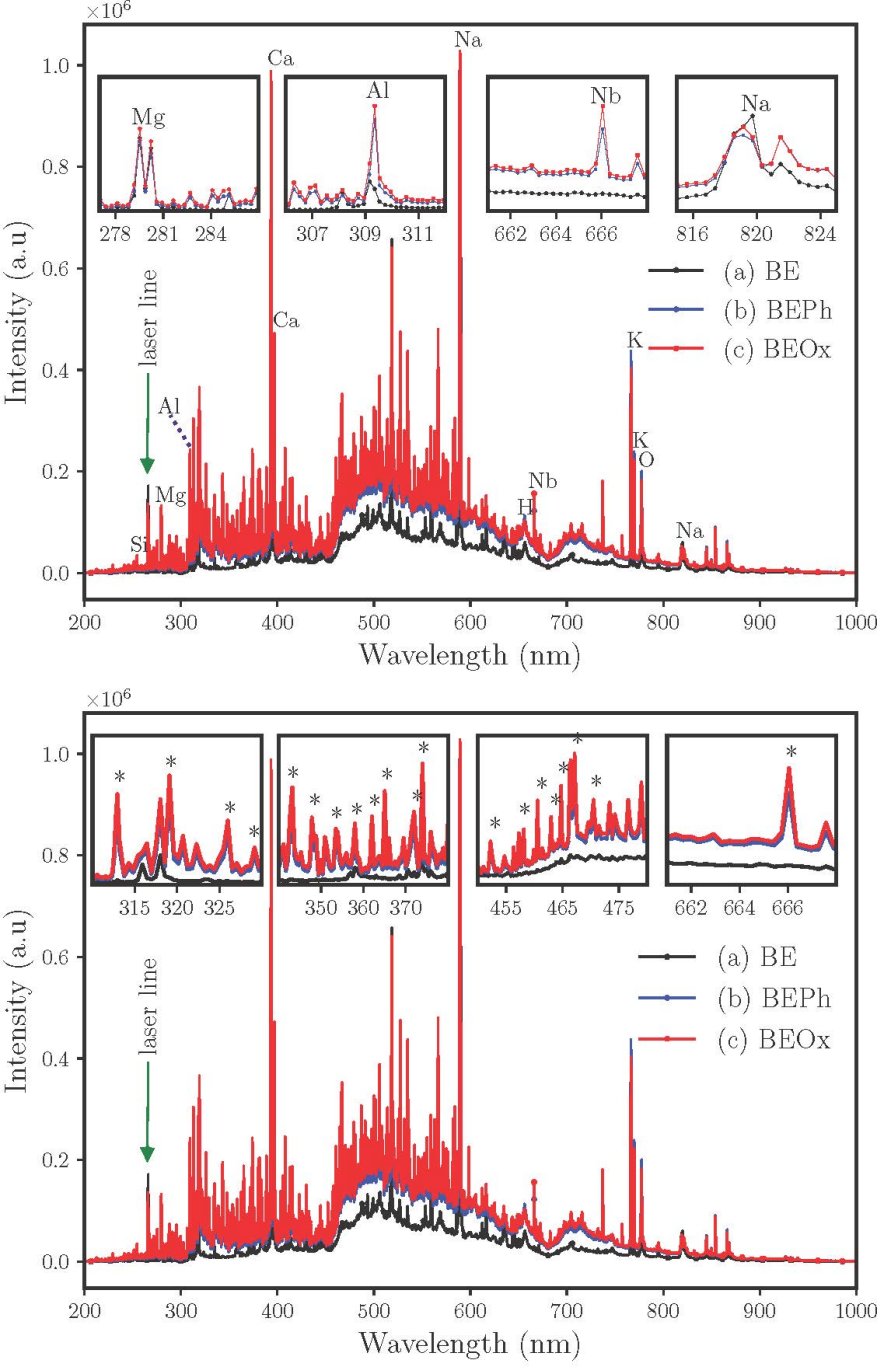
Laser-induced breakdown spectroscopy analysis for the samples BE (a), BEPh(b) and BEOx (c).

Figure 6 shows LIBS spectra for the samples BE, BEPh, and BEOx. It is observed that the samples containing niobium show a higher density of spectral lines. For the sample BE, the principal emissions lines for Mg^2+^, Al^3+^, Na^+^, at around 279 nm, 309 nm and 589 nm, respectively, are consistent with the montmorillonite structure, and these results are coherent with XPS composition in Table 1. The modification of the clay samples with niobium was characterized by the presence of many other lines throughout the spectrum region, which highlighted the lines at 666 nm. XPS analysis (Table 1), as well as the XRD results (Figure 2), suggest that the niobium compounds (NbOPO_4_ and Nb_2_O_5_) were exchanged with Na^+^ ions of the clay, due to the decreased percentage of these elements in XPS composition as observed in Figure 5. The LIBS results showed the presence of lines of Mg and Na for the BE sample, at around 280 nm and 819 nm, respectively. As demonstrated in Table 1, the compositions of Na were reduced when the BE sample was modified with niobium compounds.

The average particle size for the samples BE, NbPh, and NbOx were 12, 410, and 54 μm, respectively, and it is present in Supporting Information File 1, Figure S1. The BEPh and BEOx samples presented different particle size distributions, so the average particle size (D50) for them was 4.52 and 86 μm, respectively. These values indicate that the clay aggregates are dissociated and well dispersed, as shown in the study by Yang et al. [20].

**Figure 7:**
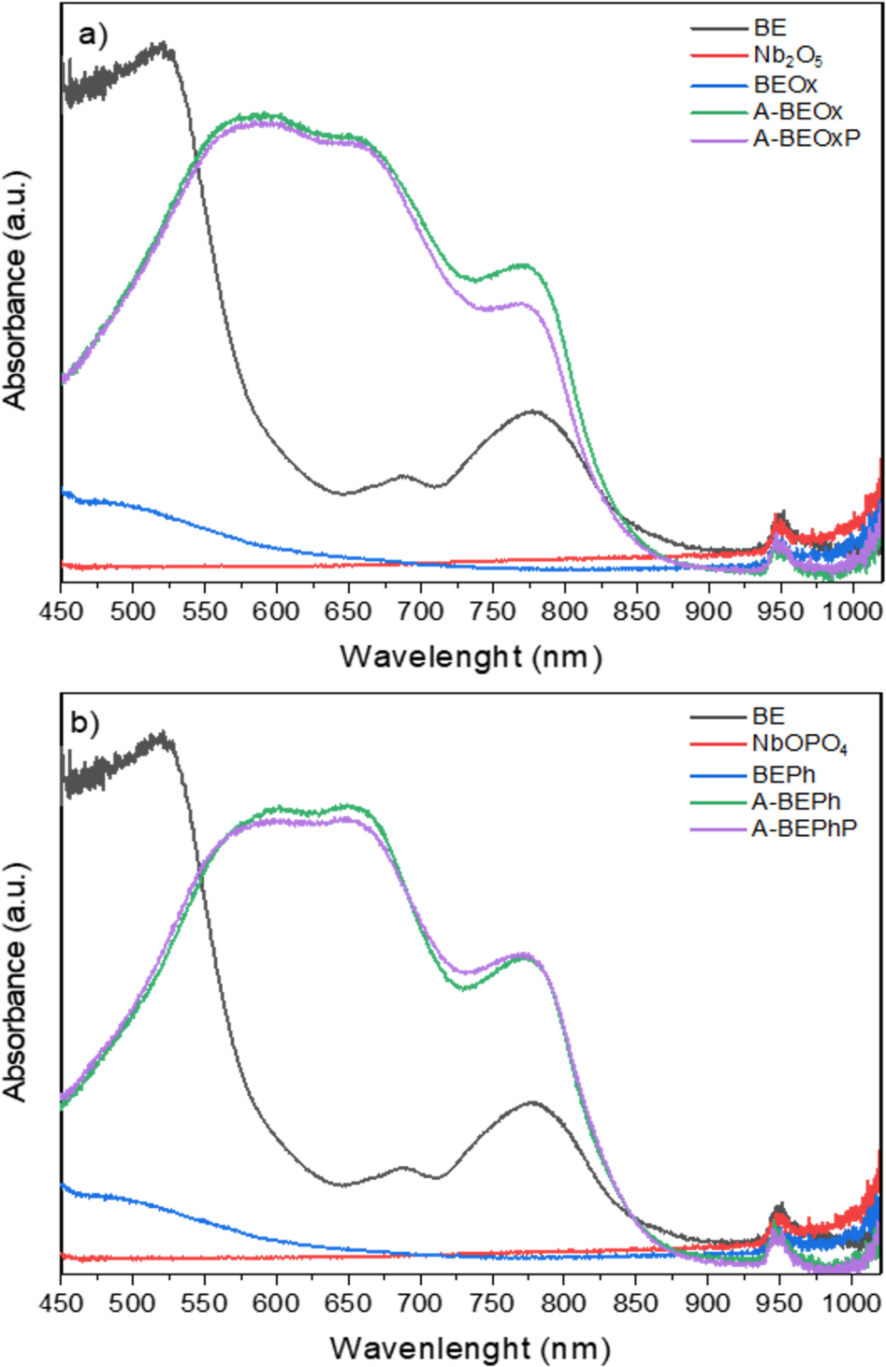
The absorption spectra in the visible region for the samples of bentonite and their modifications with niobium oxide (a) and niobium phosphate(b).

The absorbance spectra profiles in the visible region for the bentonite samples and bentonite modified with niobium are presented in Figure 7. It can be observed that the NbOPO_4_ and Nb_2_O_5_ powder samples do not show an absorption band. However, the bentonite modified with these compounds exhibited a band with a maximum of 493 nm. The samples BEOx and BEPh obtained after adsorption/photocatalysis present profiles like BE clay, with intense absorption in the UV region with a sharp drop of around 550 nm [8]. This fact indicates the feasibility of activating the A-BEPh, A-BEPhP, A-BEOx, and A-BEOXPh samples under visible light (above 400 nm) [8].

The indirect bandgap energy values for the BE, NbOPO_4_ and Nb_2_O_5_ and the bentonite clay modified with niobium BEPh and BEOx samples were estimated by the Tauc method [21]. For this, Kubelka–Munk method was applied as observed in Supporting Information File 1, Figure S2. The bandgap value of the BE sample (3.2 eV) was slightly higher than for the NbOPO_4_ and Nb_2_O_5_ compounds (3.13 and 3.2 eV, respectively). The calculated bandgap is coherent with the literature, Nakhaei et al. (2019) [22] obtained a value of 3.6 eV for bentonite clay, and for the niobium compounds Ücker et al. 2018 obtained the values of a bandgap equal to 3.0 eV [23]. The bentonite clay samples modified with niobium (BEOx and BEPh) remained with bandgap values lower compared with the clay, 3.07 and 3.13 eV, respectively. These values are consistent with the results of the study by Ascencios et al. 2019 [8] in which obtained bandgap values to clay/Nb were around 3.0 and 3.38 eV. The small decrease in values occurred due to the bentonite clay with high bandgap values, which generated impurity energy levels above the valence band edge. This results in lower energy values required to excite charge carriers, reducing the optical band [24].

**Figure 8:**
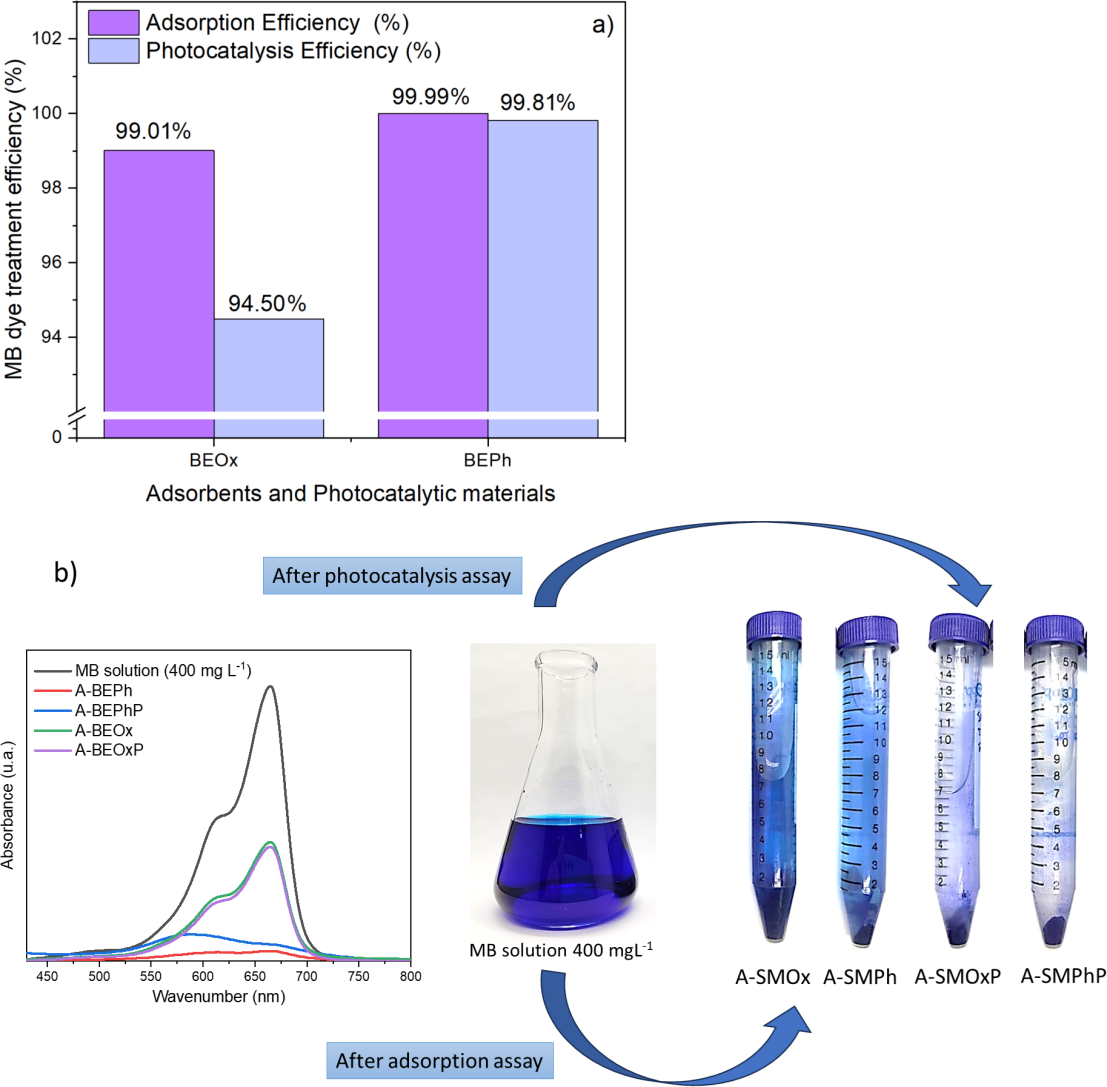
Adsorption and photocatalysis efficiency (a) and UV–vis spectra following the digital images of the MB solutions after adsorption and photocatalysis assays (b) using the starting materials BEOx and BEPh.

Figure 8 shows the results of the comparison obtained regarding the percentage of removal from adsorption and heterogeneous photocatalysis tests. The photocatalysis mechanism can be explained as follows: a semiconductor such as the BEPh and BEOx samples absorbs a photon, promoting an electron from the valence band (V_B_) to the conduction band (C_B_), creating a hole in the valence band (h_BV_^+^) [8]. These holes induce the oxidative decomposition of organic molecules adsorbed on the catalytic surface. They also react with water molecules, producing the hydroxyl radical (OH^•^). This radical rapidly attacks the dye molecules in the solution, leading to mineralization into CO_2_ and H_2_O [8].

According to the observed results, the BEOx and BEPh samples notably exhibited higher efficiency in the adsorption process (99.01% and 99.99%, respectively) than in photocatalysis. This phenomenon can be attributed to the negative surface charge of the modified clays BEOx and BEPh, which exhibit a strong affinity with the positively charged structure of the MB dye. On the other hand, the BEOx and BEPh samples demonstrated significant efficacy in MB removal, with removal rates of 94.5% and 99.81%, respectively. The preferential electron–hole (h_BV_^+^) favoring of the BEOx catalyst hindered its photocatalytic activity [8].

In a study by Asencios et al. (2019) [8], niobium-modified clay was explored for the photocatalysis of rhodamine B dye, yielding removal rates close to 95%. Additionally, Lacerda et al. (2020) [25] achieved up to 90% efficiency in the removal of reactive blue 19 dye using niobium-modified bentonite. These reports demonstrate that the material obtained in this study presents high values of MB removal under UV light, standing out as a novel material.

The absorbance profiles of the niobium-modified samples dispersed in clear paint are shown in Supporting Information File 1, Figure S3. It is possible to observe that the BEOx and BEPh samples dispersed in clear paint, despite presenting a yellow coloration, did not show absorption bands. The samples collected after the adsorption/photocatalysis assays exhibited bands in the maximum region at 664 nm, corresponding to the π → π* electronic transitions of the adsorbed MB dye [26].

Table 2 and Table 3 and Supporting Information File 1, Figures S4 and S5 present the values of the CIEL*a*b* colorimetric parameters for the samples BEOx and BEPh (powder sample, cycle) even as they disperse in colorless paint (paint sample, square), respectively. The tables also include color difference values between the samples in powder form and those dispersed in colorless paint. Colorless paint does not contain white pigment in its matrix, enhancing the dispersed pigment color. The Δ*E* parameter quantified the color difference between two samples, BEOx and BEPh, in powder form and those dispersed in colorless paint. As observed, the results of Δ*E* show that the samples A-BEPhP (12.17) and BEOx (9.82) before the adsorption/photocatalysis process when dispersed in colorless paint demonstrate a strong color parameter difference (Δ*E* = 6–12). The Δ*E* values obtained for the samples BEPh (13.62) before the adsorption/photocatalysis process, A-BEPh (23.38), A-BEOx (15.97), and A-BEOxP (13.80) were above 12, indicating a very strong color parameter difference [27].

**Table 2:** Colorimetric parameters obtained by the CIEL*a*b* system for the BEPh samples before and after the adsorption/photocatalysis assays in powder form (circle) and dispersed in colorless paint (square).

Sample	Colorimetric parameters						Images of the sample

	L*	a*	b*	C*	h	Δ*E*	

BEPh	77.02	2.47	10.38	10.67	76.67	–	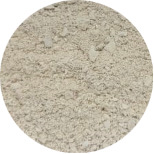
BEPh	90.54	2.19	12.03	12.23	68.01	13.62	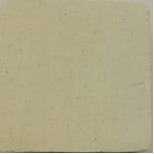

A-BEPh	13.45	4.91	−36.34	36.67	277.69	–	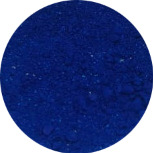
A-BEPh	32.05	3.15	−22.29	22.51	278.05	23.38	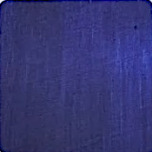

A-BEPhP	31.91	−5.08	−21.03	21.63	256.42	–	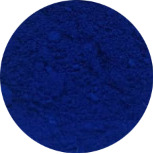
A-BEPhP	32.98	5.97	−26.01	26.68	282.92	12.17	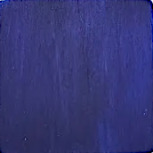

**Table 3:** Colorimetric parameters obtained by the CIEL*a*b* system for the BEPh samples before and after the adsorption/photocatalysis assays in powder form (circle) and dispersed in colorless paint (square).

Sample	Colorimetric parameters						Images of the sample

	L	a*	b*	C*	h	Δ*E*	

BEOx	80.96	3.07	13.17	11.05	73.85	–	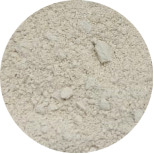
BEOx	90.42	0.71	12.03	9.63	85.78	9.82	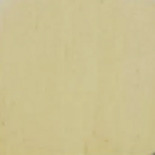

A-BEOx	22.91	13.94	−36.80	39.55	290.74	–	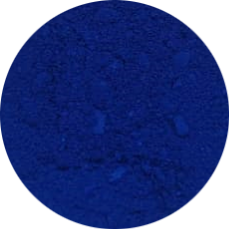

A-BEOx	30.93	6.79	−24.99	25.90	285.20	15.97	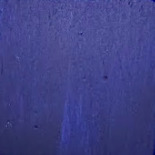

A-BEOxP	18.25	7.90	−30.06	31.00	284.72	–	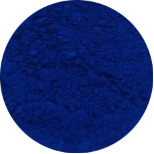

A-BEOxP	30.65	7.15	−24.06	25.10	286.54	13.80	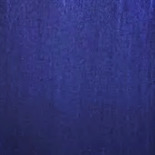

The colorimetric parameters presented in Table 2 and Table 3 and in Supporting Information File 1, Figures S2 and S3 demonstrate that the test specimens painted with niobium-modified clay before the adsorption/photocatalysis of MB show higher luminosity (L). When the clay samples were colored MB, after the adsorption/photocatalysis process, the luminosity decreased. The A-BEPhP sample shows a slight tendency towards green coloration (-a*). On the other hand, when evaluating the b* parameter, it is observed that all samples obtained after adsorption/photocatalysis of MB (A-BEPh, A-BEPhP, A-BEOx and A-BEOxP), whether in powder form or dispersed in paint, show a tendency towards blue coloration, as observed in the negative b* values.

The BEPh, BEOx, A-BEPh, A-BEOx, A-BEPhP, and A-BEOxP samples were evaluated for their in vitro antibacterial capacity using the minimum inhibitory concentration (MIC) method against the pathogenic bacteria *Proteus mirabilis* and *Bacillus cereus*. The results obtained are presented in Table 4.

**Table 4:** MIC (mg/mL) for the in vitro antibacterial activity in water.

Sample	BEPh	BEOx	A-BEPh	A-BEOx	A-BEPhP	A-BEOxP

MIC *P. mirabilis*	–^a^	–^a^	0.31	0.31	0.15	0.62

^a^MIC values were not determined.

The samples A-BEPh, A-BEPhP, A-BEOx, and A-BEOxP demonstrated antibacterial activity against the *Proteus mirabilis* bacterial strains, with MIC values recorded at 0.31, 0.15, 0.31, and 0.62 mg/mL, respectively. In contrast, the BEPh and BEOx samples exhibited no inhibitory effects at the tested concentrations for this microorganism.

Observing the adsorption and photocatalysis efficiency in Figure 8a, similar values for the samples BEPhP and BEPh were obtained (99.81% and 99.81%, respectively) that is, the amount of dye present in these samples is practically the same. The MIC results of these samples indicated 0.15 mg/mL and 0.31 mg/mL for the samples A-BEPhP and A-BEPh, respectively. The adsorption and photocatalysis efficiency for the samples BEOxP and BEOx were 94.50% and 99.01%, respectively. These results suggest that sample A-BeOX contains a larger amount than sample A-BePh. The A-BEOxP sample yielded an MIC of 0.62 mg/mL, while the A-BEOx had an MIC of 0.31 mg/mL, indicating a more effective antibacterial action against the tested strains.

It is noteworthy that antibacterial activity was only observed in materials that had the addition of methylene blue, highlighting the influence of the dye on the assay outcomes. According to Thesnaar (2021) [28], many studies have demonstrated that methylene blue, either alone or in combination with other compounds, possesses antibacterial activity; however, the precise mechanism of action remains unclear. The BEPh, BEOx, A-BEPh, A-BEOx, A-BEPhP, and A-BEOxP samples did not exhibit minimum inhibitory activity against the other studied bacterial strain *Bacillus cereus*.

These results highlight the selectivity of the studied samples (A-BEPh, A-BEPhP, A-BEOx, and A-BEOxP) in inhibiting Gram-negative bacteria, which typically exhibit increased resistance due to the presence of an outer membrane that protects them from certain antimicrobial agents [29]. Therefore, the preliminary results indicate that bentonite clay modification with niobium oxide (BEOx) and niobium phosphate (BEPh), when adsorbed and/or photocatalyzed for the treatment of wastewater containing MB, demonstrates promising selective antibacterial activity against Gram-negative bacteria.

## Conclusion

In conclusion, the presented study demonstrates that the modification of bentonite clay with niobium phosphate and niobium oxide can be achieved through a simple and low-cost procedure. The XRD results revealed that the raw bentonite contains additional phases such as kaolinite and quartz. The niobium incorporated into bentonite clay (BEPh and BEOx) indicated that the niobium modification did not destroy the structure of the montmorillonite layer, as indicated by the XRD results. Furthermore, analyses using XPS and LIBS confirmed that the niobium compounds exchanged with Na^+^ ions of the clay tetrahedral sheet.

The bentonite sample, along with the BEPh and BEOx samples, exhibited adsorption and photocatalytic efficiencies exceeding 94% for the removal of MB in water. This process resulted in a final blue-colored product as evidenced by the colorimetric parameters obtained through the CIEL*a*b* color space. The samples derived from the adsorption/photocatalysis tests (A-BEPh, A-BEPhP, A-BEOx, and A-BEOxP) demonstrate significant antibacterial activity against the Gram-negative bacteria *Proteus mirabilis*, highlighting the influence of the dye in the assay outcomes.

The implications of this work extend to the development of a novel hybrid pigment. This pigment, synthesized from abundant natural clays of the Guarapuava region in conjunction with niobium, an abundant metal in Brazil, is easily synthesized, cost-effective, and has potential to be used as a pigment in commercial paints. Moreover, it exhibits antibacterial properties against pathogens responsible for various diseases, including ocular and ear infections.

## Supporting Information

File 1 Additional figures.

## Data Availability

Data generated and analyzed during this study is available from the corresponding author upon reasonable request.
