# Supplementary material for: Clays enhanced with niobium: potential in wastewater treatment and reuse as pigment with antibacterial activity
Source: Beilstein J Nanotechnol. 2025 Feb 10;16:141–54. doi: 10.3762/bjnano.16.13 (PMC11833176; doi:10.3762/bjnano.16.13)
Supplement: File 1 — Additional figures. [file Beilstein_J_Nanotechnol-16-141-s001.pdf]

BEILSTEIN JOURNAL OF NANOTECHNOLOGY

# Supporting Information

for

# Clays enhanced with niobium: potential in wastewater treatment and reuse as pigment with antibacterial activity

Silvia Jaerger, Patricia Appelt, Mario Antônio Alves da Cunha, Fabián Ccahuana Ayma, Ricardo Schneider, Carla Bittencourt and Fauze Jacó Anaissi

*Beilstein J. Nanotechnol.* **2025,** *16,* 141–154. doi:10.3762/bjnano.16.13

# Additional figures

License and Terms: This is a supporting information file under the terms of the Creative Commons Attribution License (https://creativecommons.org/licenses/by/4.0). Please note that the reuse, redistribution and reproduction in particular requires that the author(s) and source are credited and that individual graphics may be subject to special legal provisions.
The license is subject to the *Beilstein Journal of Nanotechnology* terms and conditions: (https://www.beilstein-journals.org/bjnano/terms)

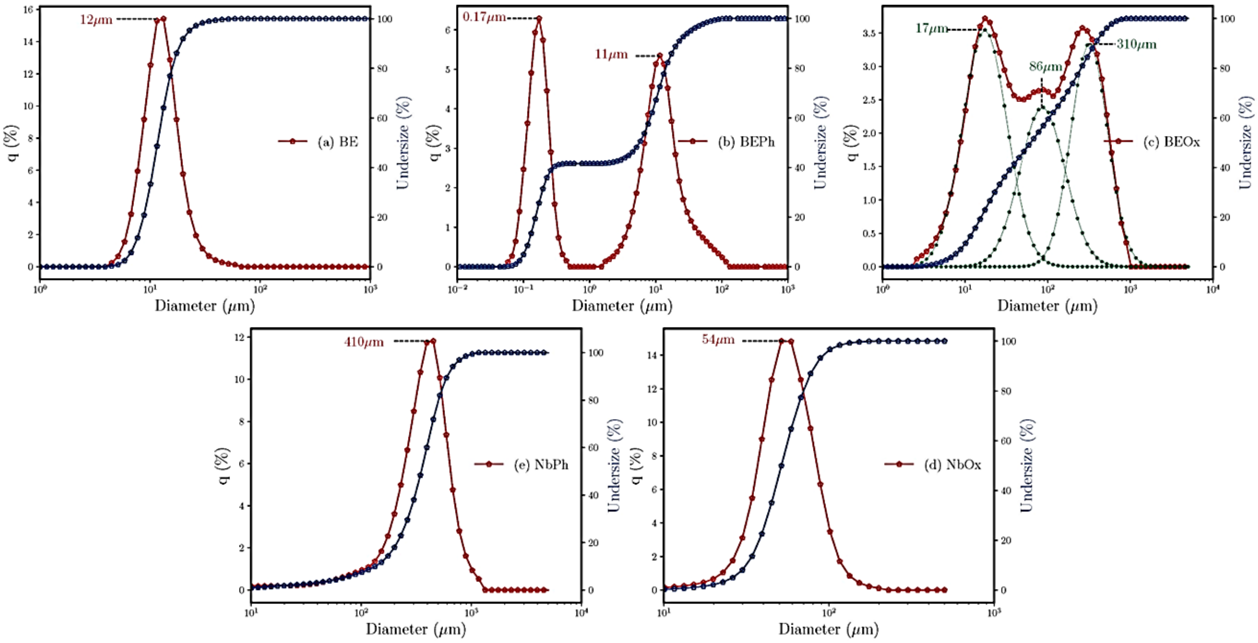


**Figure S1:** Particle size distribution for the samples BE (a), BEPh (b),BEOx (c), NbPh (d), and NbOx (e).

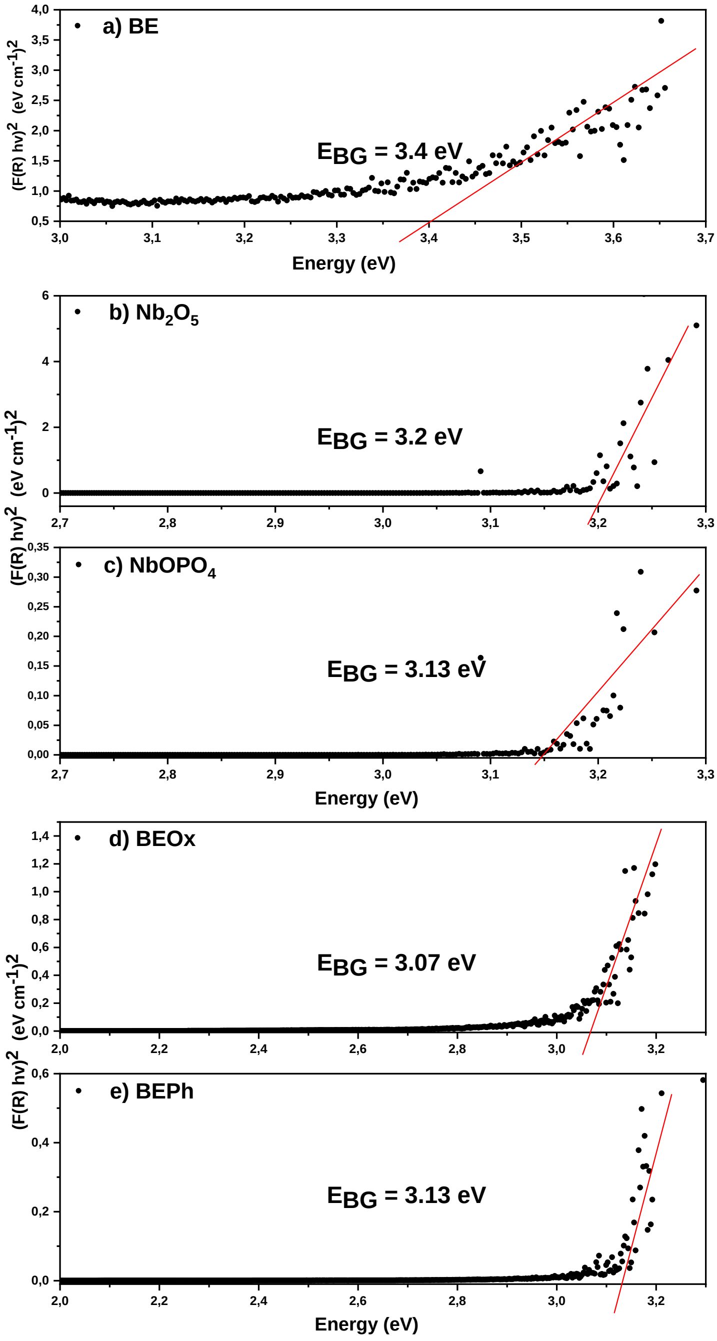


**Figure S2:** Kubelka–Munk spectra of the samples BE (a), $Nb_2O_5$ (b), $NbOPO_4$ (c), BEOx (d), and BEPh (e).

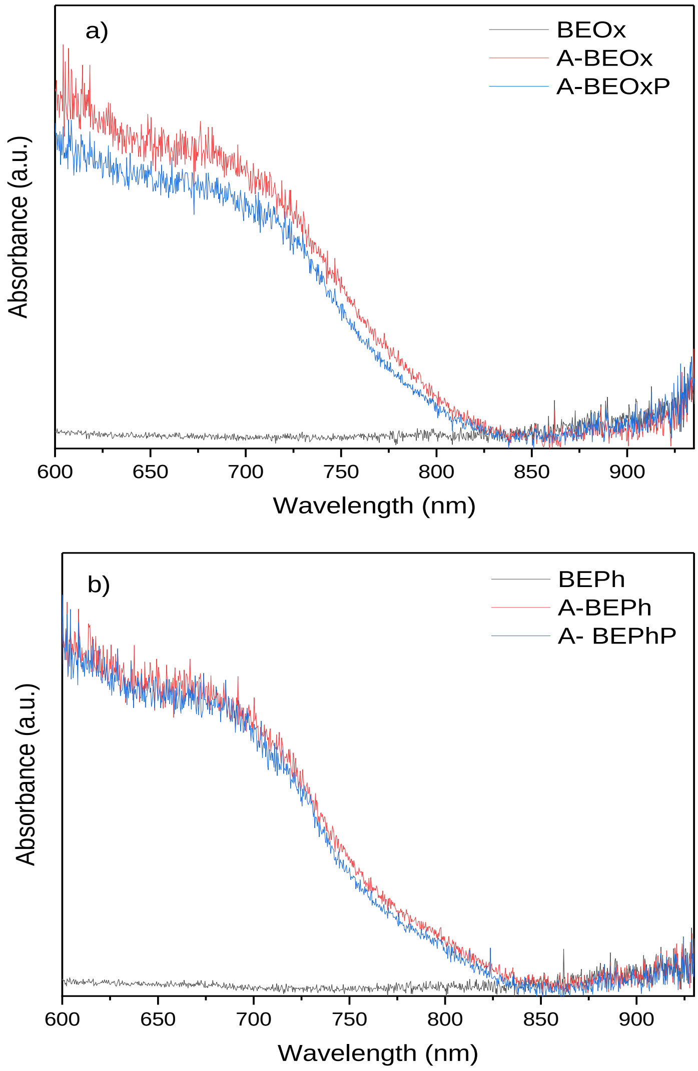


**Figure S3:** Absorbance spectra in the visible region for the bentonite samples modified with niobium oxide (a) and niobium phosphate (b) dispersed in colorless paint.

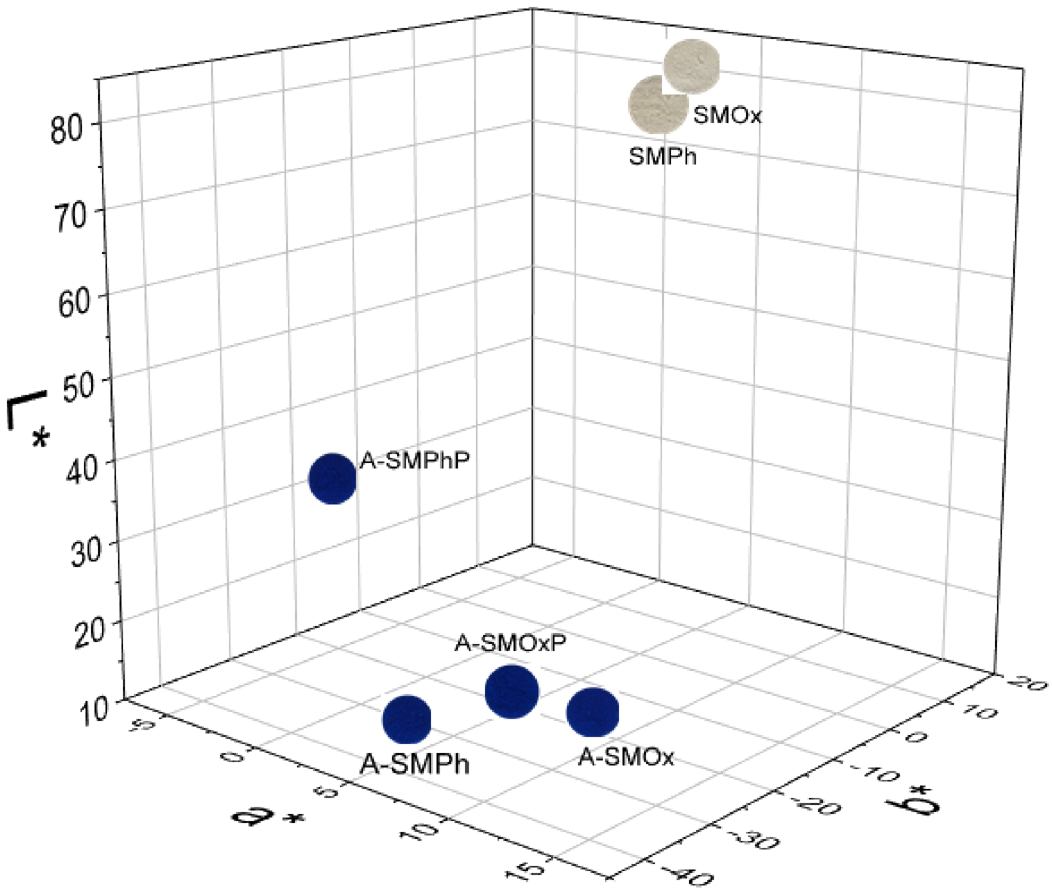


**Figure S4:** A graph of the CIEL*a*b* system highlighting the colorimetric changes of the BEPh and BEOx samples, obtained before and after the adsorption/photocatalysis process of MB in powder form.

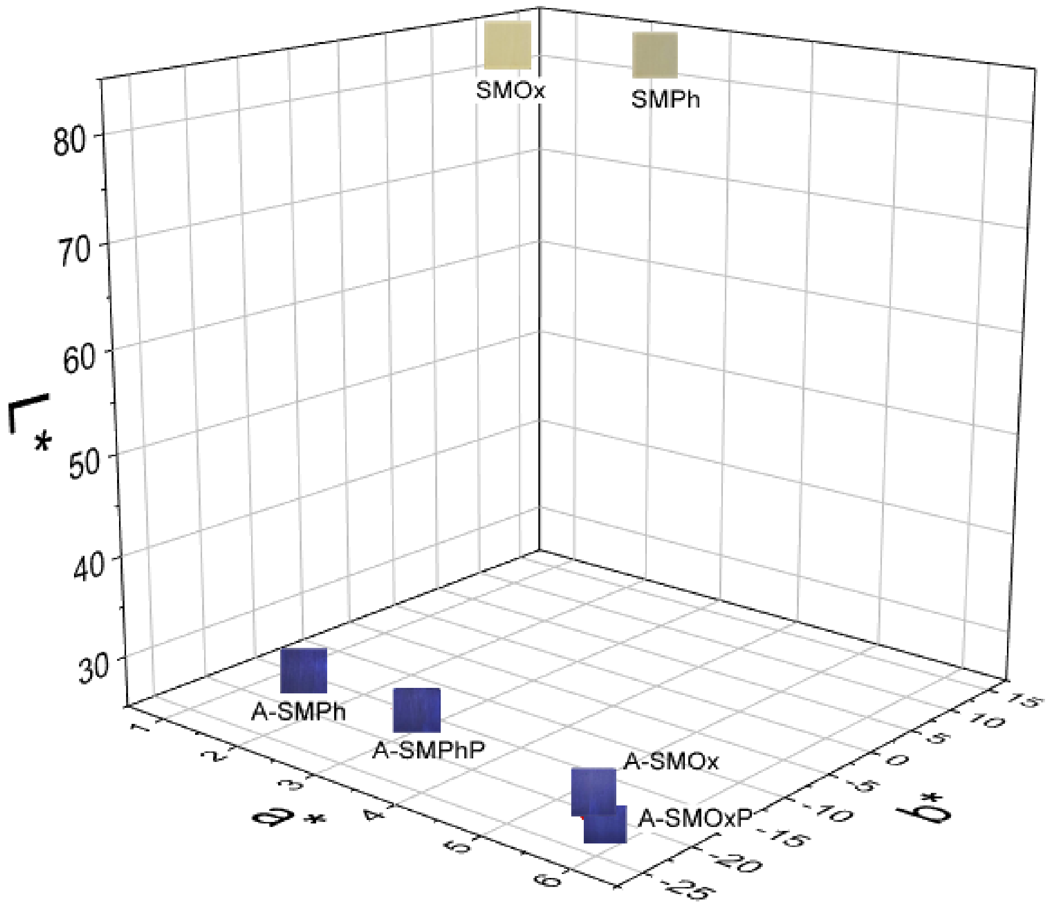


**Figure S5:** A graph of the CIEL*a*b* system highlighting the colorimetric changes of the BEPh and BEOx samples, obtained before and after the adsorption/photocatalysis process of MB as a pigment dispersed in clear paint.
